# Molecular Mechanism of the Effect of Zhizhu Pill on Gastroesophageal Reflux Disease Based on Network Pharmacology and Molecular Docking

**DOI:** 10.1155/2022/2996865

**Published:** 2022-05-19

**Authors:** Jinke Huang, Yitian Wang, Peng Xu, Jiali Liu, Jinxin Ma, Yu Wang, Zhihong Liu, Mi Lv, Fengyun Wang, Xudong Tang

**Affiliations:** ^1^Department of Gastroenterology, Xiyuan Hospital, China Academy of Chinese Medical Sciences, Beijing, China; ^2^Institute of Clinical Basic Medicine of Chinese Medicine, China Academy of Chinese Medical Sciences, Beijing, China; ^3^Department of Gastroenterology, Peking University Traditional Chinese Medicine Clinical Medical School (Xiyuan), Beijing, China; ^4^School of Traditional Chinese Medicine, Beijing University of Chinese Medicine, Beijing, China; ^5^Institute of Gastroenterology, Xiyuan Hospital, China Academy of Chinese Medical Sciences, Beijing, China

## Abstract

**Background:**

To investigate the pharmacological mechanism of Zhizhu pill (ZZP) against gastroesophageal reflux disease (GERD), network pharmacology in combination with molecular docking was applied in this study.

**Methods:**

Active compounds of ZZP and target genes related to GERD were identified through public databases. Subsequently, the obtained data were used as a basis for further network pharmacological analysis to explore the potential key active compounds, core targets, and biological processes involved in ZZP against GERD. Finally, the results predicted by network pharmacology were validated by molecular docking.

**Results:**

Twenty active components of ZZP were identified to act on 59 targets related to GERD. Enrichment analysis revealed that multiple biological processes including response to oxygen levels, response to oxidative stress, and response to reactive oxygen species were involved in the GERD ZZP treatment with ZZP. ZZP had an impact on the prognosis of GERD mainly through the HIF-1 signaling pathway, PI3K-Akt signaling pathway, and pathways in cancer. Further analysis identified the key components and core targets of ZZP against GERD, of which nobiletin, didymin, luteolin, and naringenin were key components, and PPARG, MMP9, JUN, TP53, PTGS2, EGFR, MAPK3, CASP3, AKT1, and VEGFA were the core targets. Molecular docking verified the stable bonds formed between the key components and the core targets.

**Conclusions:**

The results of this study predict that the therapeutic effects of ZZP in GERD are mediated at least in part via PPARG, MMP9, JUN, TP53, PTGS2, EGFR, MAPK3, CASP3, AKT1, and VEGFA. These results may be useful in providing an experimental basis and new ideas for further research on ZZP in GERD.

## 1. Introduction

Gastroesophageal reflux disease (GERD) is a gastrointestinal motility disorder in which gastric contents reflux into the esophagus or oral cavity leading to symptoms or complications, with heartburn and regurgitation as typical symptoms [1, 2]. In United States, the prevalence of GERD is estimated to be 19.8% [3], and the annual cost of managing GERD amounts to $15–20 billion [4], which imposes a heavy medical and economic burden on society. Furthermore, long-term GERD can lead to esophageal inflammation and esophageal cellular changes, thus increasing the risk of developing esophageal cancer [1]. The first-line medical treatment for GERD are proton pump inhibitors (PPIs) [5]. However, due to the complex pathophysiological mechanism of GERD, the use of PPIs does not achieve the expected effect [6–8]. As a result, complementary and alternative therapies are gradually gaining interest [9].

Chinese herbal medicine (CHM) has been widely used for symptom management of GERD in China [10]. Zhizhu pill (ZZP), an ancient classical formula consisting of two herbs (zhishi (Aurantii Fructus Immaturus) and baizhu (*Atractylodes macrocephala* Koidz)), is originated from Shang Han Zabing Lun (200–210, AD) and has been widely used for the treatment of functional gastrointestinal diseases. Evidence confirmed that the ZZP was beneficial for GERD treatment [11]. Nevertheless, there is no literature expounding the underlying therapeutic mechanism of ZZP so far.

**Figure 1:**
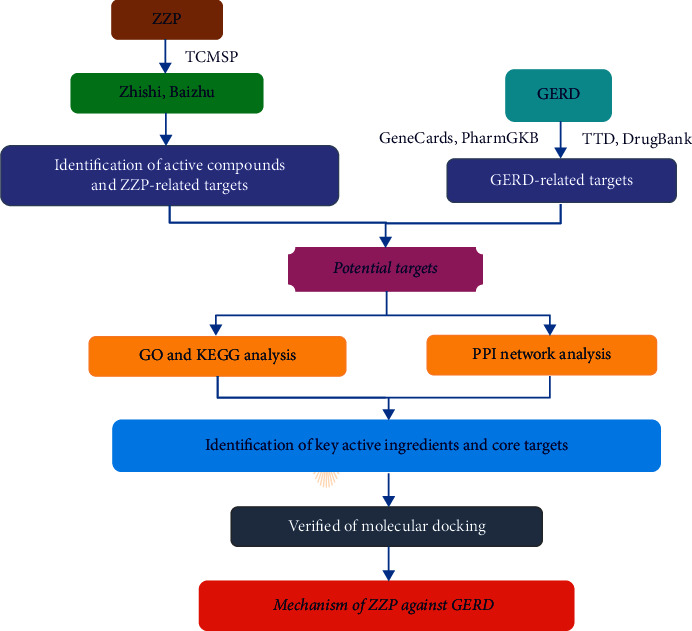
Workflow of the study.

Due to the “multicomponent” and “multitarget” characteristics of CHM formula, it is difficult for traditional experimental methods to reveal the comodular association mechanism of CHM-component-gene and disease. Network pharmacology is an innovative approach to elucidate the synergy and potential mechanisms of component-target and target-disease networks [12–14], and it provides a new perspective on the therapeutic mechanisms of CHM formula [14]. Recently, in silico techniques were used to decode disease targets and the development of novel drugs, and valuable discoveries have been made. Therefore, to uncover the mechanism of ZZP for GERD, network pharmacology in combination with molecular docking was applied in this study [15]. Workflow of the present study is detailed in Figure 1.

## 2. Methods

### 2.1. Screening of Active Compounds and Targets

The Traditional Chinese Medicine Systems Pharmacology (TCMSP) [16] was applied to identify the compounds and targets of Aurantii Fructus Immaturus, and *Atractylodes macrocephala* Koidz. Oral bioavailability ≥30% and drug-like ≥0.18 were identified as criteria for screening drug compounds [17]. UniProt [18] was used to normalize gene symbols for acquiring targets.

### 2.2. Identification of Target Genes Related to GERD

DrugBank (https://www.drugbank.ca/), TTD (https://bidd.nus.edu.sg/group/cjttd/), PharmGKB (https://www.pharmgkb.org/), and GeneCards (https://www.genecards.org/) were applied to identify target genes related to GERD. A correlation score ≥10 was established as a screening criterion for GeneCards target genes [19].

### 2.3. Drug-Compound-Target Network Analysis

The common targets of drugs and diseases were obtained through a Venn diagram, and the overlapping results were considered as potential targets for ZZP treatment of GERD. To further explore the correlation between drugs and diseases, the drug-compound-target network was constructed with Cytoscape 3.7.2 software [20].

### 2.4. GO and KEGG Enrichment Analysis

To further explore the biological process of ZZP against GERD, GO and KEGG enrichment analysis were carried out with clusterProfiler package [21] in R 4.0.5 software. *P* < 0.05 was regarded as the criterion for statistical differences.

### 2.5. PPI Analysis and Core Targets Identification

PPI analysis was performed through STRING (https://string-db.org/) with interaction score as 0.400 [22]. The Cytoscape plugin cytoHubba [23] was applied to identify core targets by calculating degree centrality, closeness centrality, betweenness centrality, network centrality, eigenvector centrality, and local average connectivity.

### 2.6. Verification through Molecular Docking

Based on the core targets of ZZP against GERD that have been identified by cytoHubba, molecular docking was performed with Discovery Studio 2019 to validate the compound-target correlation. The structures of key compounds and core macromolecular protein target receptors related to GERD were downloaded from PubChem (https://pubchem.ncbi.nlm.nih.gov/) [24] and RCSB PDB (https://www.rcsb.org/) [25], respectively. LibDock docking conditions were as follows: docking preference was set to high quality, conformational method was set to FAST, and other parameters were set to default values. In the context of the parameters based on the above settings, the optimal binding site for each protein automatically identified by Discovery Studio 2019 was docked to the corresponding molecule. A higher LibDock score suggested a more plausible prediction of target binding activity.

## 3. Results

### 3.1. Active Compounds and Targets

Through TCMSP, 65 compounds of Aurantii Fructus Immaturus and 55 compounds of *Atractylodes macrocephala* Koidz were identified, respectively. According to the oral bioavailability ≥30% and drug-like ≥0.18, 21 active compounds of ZZP were identified finally, of which 17 belonged to Aurantii Fructus Immaturus and 4 to *Atractylodes macrocephala* Koidz. Furthermore, 117 targets corresponding to these 21 active compounds were identified. Details of the 21 active compounds and 117 targets are presented in Supplement A.

### 3.2. Targets Associated with GERD

By searching databases, 1613 target genes related to GERD were obtained, of which 1283 were downloaded from the GeneCards, 200 from PharmGKB, 118 from DrugBank, and 12 from TTD. After removing the duplicates, 1476 target genes related to GERD were finally obtained. 1476 target genes are detailed in Supplement B.

### 3.3. Network Construction

**Figure 2:**
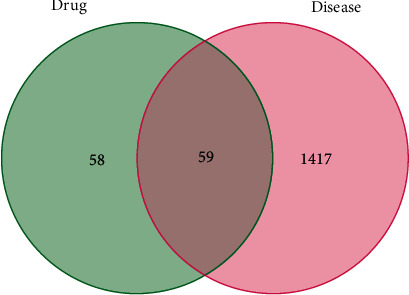
Venn diagram of targets from ZZP and GERD.

**Figure 3:**
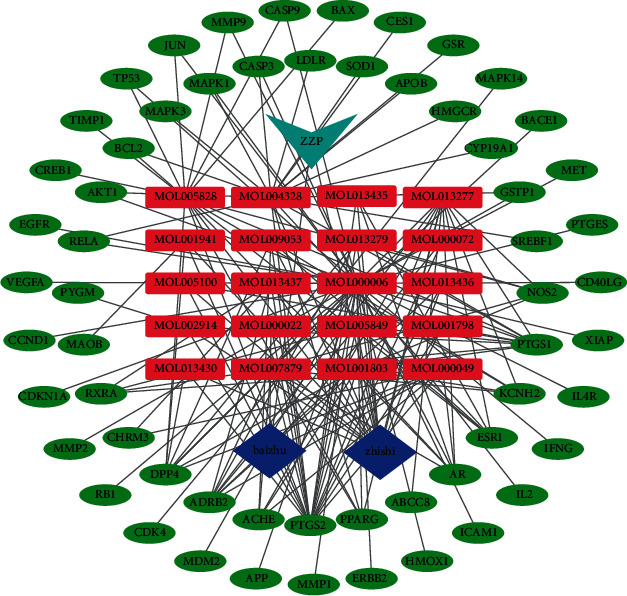
Drug-compound-target gene network of ZZP. The red squares represent the components; the green ellipses represent targets; purple diamonds represent different herbs; and blue arrows represent ZZP. The edges represent the relationship between the components and the targets.

The Venn diagram (Figure 2) identifies 59 overlapping targets for drugs and diseases. A drug-compound-target gene network was constructed based on the identified overlapping targets. As shown in Figure 3, this network included 20 components, 59 targets, 82 nodes, and 167 edges.

### 3.4. Enrichment Analysis of GO and KEGG

**Figure 4:**
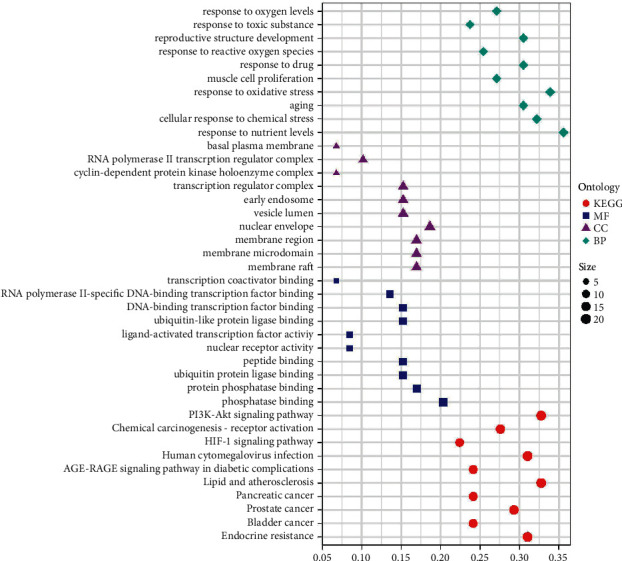
Results of GO and KEGG enrichment analysis.

According to the results of enrichment analysis, the biological processes were mainly enriched in response to oxygen levels, response to oxidative stress, response to reactive oxygen species, response to nutrient levels, cellular response to chemical stress, aging, muscle cell proliferation, response to drug, reproductive structure development, and response to toxic substance. With KEGG analysis, pathways related to cancer, endocrine resistance, lipid and atherosclerosis, AGE-RAGE signaling pathway in diabetic complications, human cytomegalovirus infection, HIF-1 signaling pathway, and PI3K-Akt signaling pathway were most significantly enriched. More details of the enrichment results are presented in Figure 4 and Table 1.

### 3.5. PPI Network and Core Subnetwork

**Figure 5:**
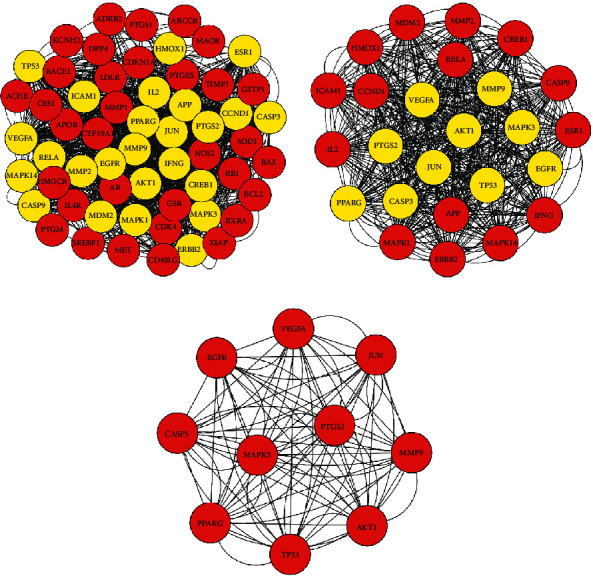
Process of topological screening for the PPI network. (a) PPI network from STRING visualized with Cytoscape. (b) PPI network of more significant proteins extracted from (a) by filtering 6 parameters: BC, CC, DC, EC, NC, and LAC. (c) Core PPI network of core targets extracted from (b).

With the PPI network constructed by STRING, 58 nodes and 1250 interactions were observed. The first screening by cytoHubba yielded a network of 25 nodes and 542 interactions, and the second screening yielded a dense region network with 10 nodes and 90 interactions. PPARG, MMP9, JUN, TP53, PTGS2, EGFR, MAPK3, CASP3, AKT1, and VEGFA were identified as core targets. Details are presented in Figure 5.

## 4. Results of Validation

**Figure 6:**
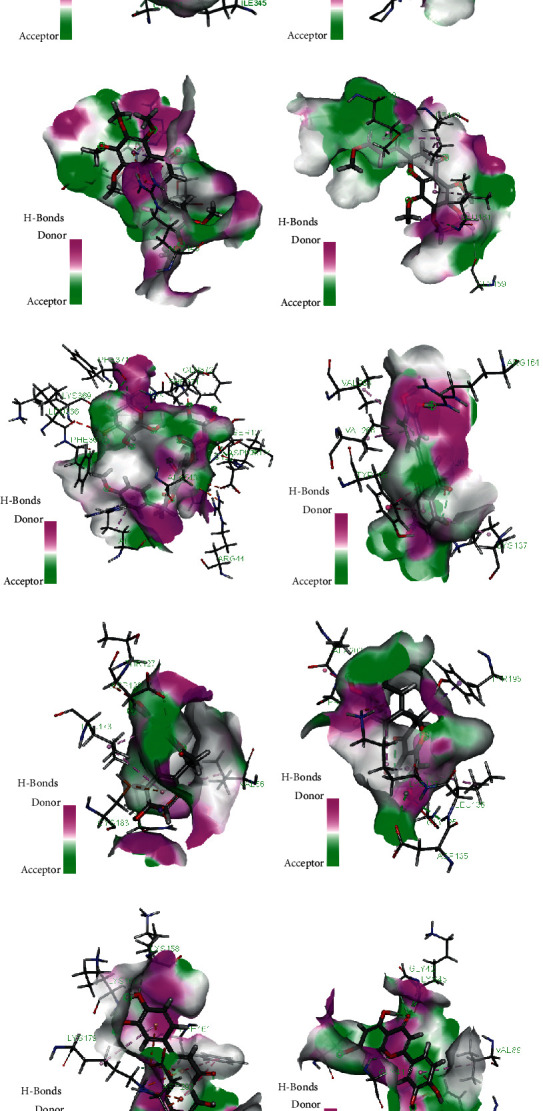
3D molecular docking model. (a) PPARG; (b) MMP9; (c) JUN; (d) TP53; (e) PTGS2; (f) EGFR; (g) MAPK3; (h) CASP3; (i) AKT1; and (j) VEGFA.

**Figure 7:**
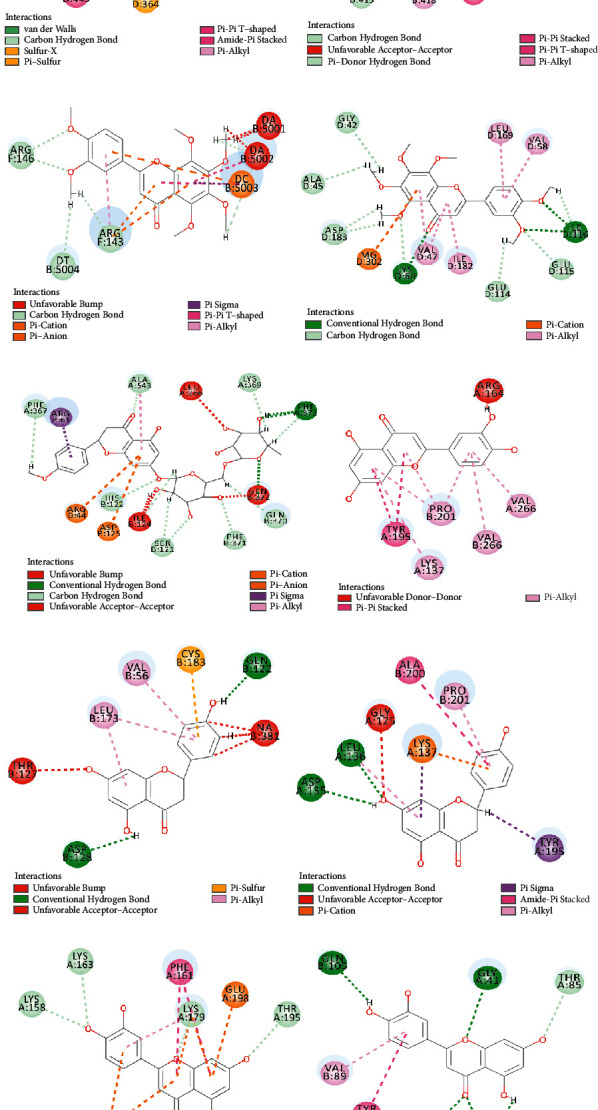
2D molecular docking model. (a) PPARG; (b) MMP9; (c) JUN; (d) TP53; (e) PTGS2; (f) EGFR; (g) MAPK3; (h) CASP3; (i) AKT1; and (j) VEGFA.

Molecular docking was performed based on the ten core targets and the four corresponding key active compounds. According to the results of molecular docking, all LibDock scores over “80” (Table 2), suggesting that all key active ingredients were well docked to the corresponding targets. 3D and 2D molecular docking models are presented in Figures 6 and 7, respectively.

## 5. Discussion

GERD is among the most frequent reasons for outpatient gastroenterology consultation [36]. The first-line drugs currently recommended for GERD treatment are PPIs; however, the efficacy has not met expectations [6]. ZZP has been widely used to treat GERD in China with definite benefits, but the pharmacological mechanism has not been elucidated. To uncover the pharmacological mechanism of ZZP against GERD, network pharmacology in combination with molecular docking was, therefore, applied in this study.

Through public databases, 20 active compounds of ZZP were found to act on 59 target genes related to GERD, which further validated the “multicomponent” and “multitarget” characteristics of ZZP. Based on these findings, it was reasonable to adopt a network pharmacology approach to elucidate the component-target and target-disease network synergies and intrinsic mechanism of ZZP for GERD treatment. To the best of our knowledge, this was the first study to use a network pharmacology approach to reveal the molecular mechanism of ZZP for GERD treatment.

With network pharmacology, multiple biological processes including response to oxygen levels, response to reactive oxygen species, and response to oxidative stress were found to be involved in the treatment of GERD with ZZP. These findings suggested that ZZP might play a role in the treatment of GERD mainly by regulating oxidative stress. A new view of the pathogenesis of GERD was recently reported, describing it as an inflammatory disease characterized by increased production of cytokines, chemokines, and reactive oxygen species (ROS), as well as disruption of the endogenous antioxidant defense system [22]. The formation of ROS and inflammation play an important role in GERD pathogenesis, and often go hand in hand [37]. In the esophageal mucosa of GERD patients, increased chemiluminescence, peroxide, and superoxide dismutase have been observed [38]. Moreover, abnormal changes in oxidative stress markers were observed in the esophageal mucosa of patients with esophagitis, heterogeneous hyperplasia, or adenocarcinoma [39], which further confirms that oxidative stress mediates pathological changes in the esophageal mucosa. It is not difficult to reveal the reasons for the above phenomenon because ROS has an inhibitory effect on the endogenous antioxidant system and esophageal reflux often stimulates the excessive production of ROS [37]. In addition to inhibiting gastric acid secretion, PPIs also target the inflammatory response and oxidative stress in the esophageal mucosa [38]. According to KEGG analysis, ZZP have an impact on the prognosis of GERD through the PI3K-Akt signaling pathway and HIF-1 signaling pathway; both of these pathways are closely related to inflammation and oxidative stress [26, 27]. In summary, the above evidence consistently reveals that oxidative stress may be a new target for the prospective treatment of GERD, and ZZP can play a therapeutic role through this target.

Based on the findings of network pharmacology, further analysis was conducted to identify the key active compounds and core targets. Nobiletin, didymin, luteolin, and naringenin were identified as the key active compounds of ZZP against GERD. It has been found that didymin is beneficial to prevent the generation of ROS as well as lipid peroxidation products and the release of inflammatory cytokines and chemokines, thereby protecting the digestive tract [28, 29]. Therefore, didymin is regarded as a promising natural therapeutic agent with antioxidant effects. For luteolin, it has been found to reduce ROS and LOOH levels, which in turn play a role in regulating oxidative stress [30]. Moreover, it can also improve inflammation by decreasing the levels of TNF, IL-1*β*, and IL-6 and increasing the levels of IL-4 and IL-10 [30]. For naringenin, its antioxidant effects are mainly attributed to the promotion of free radical reduction and enhancement of antioxidant activity [31]. Recent evidence suggests that naringenin supplementation also helps to suppress cytokine expression, which in turn prevents intestinal barrier defects [32]. Thus, the key components predicted in this study have antioxidant and inflammatory modulating effects, and these findings are supported by early evidence.

PPARG, MMP9, JUN, TP53, PTGS2, EGFR, MAPK3, CASP3, AKT1, and VEGFA were predicted as the core targets of ZZP against GERD. For PPARG, it belongs to oxidative stress genes and is involved in the regulation of ROS production and inflammatory responses [33]. MMP9 is involved in the breakdown of extracellular matrix during normal physiological processes and is closely associated with tissue damage/repair [34]. It is well known that GERD is defined as symptoms or tissue damage caused by abnormal reflux of stomach contents into the esophagus [35]. Significant FUN expression was observed in esophageal cells stimulated by DNA microarray in a low pH environment, indicating that JUN may play an important role in the development of GERD [40]. It may therefore be a potential target for the treatment of GERD. Tp53 is a tumor suppressor gene that, once mutated, would promote GERD to esophageal adenocarcinoma [41]. PTGS2, a protein-coding gene involved in regulating inflammation, has become a therapeutic target for many inflammatory diseases [42, 43] and is therefore expected to be a new therapeutic target for GERD. EGFR plays an important role in epithelial repair, and patients with GERD have been found to have lower EGFR expression levels than patients with Barrett's esophagus or esophageal adenocarcinoma, indicating that EGFR expression is directly associated with disease progression [44]. MAPK3 is involved in the regulation of inflammation, and inhibitors of MAPK3 have been found to have a beneficial effect on inflammatory diseases [45]. For CASP3, it is involved in the production of reactive oxygen species and therefore has a crucial role in the regulation of oxidative stress [46]. Activation of Akt1 is associated with inflammation, oxidative stress, and accumulation of oxidized lipids, and these events form a positive feedback loop that exacerbates the consequences of oxidative stress [47]. VEGFA can stimulate endothelial cell proliferation, chemotaxis, and vascular permeability, and some studies suggest that the expression of VEGFA is closely related to inflammation and can be used as an early marker of inflammatory diseases [48]. In summary, targets predicted in this study are all involved in the regulation of oxidative stress and inflammation, and these results corroborate the feasibility of ZZP in the treatment of GERD at the molecular level.

With molecular docking analysis, all LibDock score of key components to core targets were over “80,” indicating that ZZP can effectively bind to specific proteins in GERD targets. These binding results further validate that the predicted results of network pharmacology are credible.

Limitations must be acknowledged. First, the upregulation and downregulation of predicted targets cannot be clarified by a network pharmacology approach and is therefore not conducive to an accurate understanding of the mechanism of components acting on targets. Second, only compounds of ZZP in TCMSP were analyzed, which might have caused the absence of some components and therefore limited the accuracy of the results. Third, limited by the deficiencies of systems biology, multidirectional pharmacology, computational biology, and network analysis, this study provided only preliminary predictions, and the results have not been verified in clinical and basic experiments. Thus, further pharmacological experimental validation is still necessary.

## 6. Conclusion

The results of this study predict that the therapeutic effects of ZZP in GERD are mediated at least in part via PPARG, MMP9, JUN, TP53, PTGS2, EGFR, MAPK3, CASP3, AKT1, and VEGFA. These results may be useful in providing the foundation for subsequent experimental investigation and may offer ideas for the multidimensional and multilevel research of CHM formulae.

## Figures and Tables

**Table 1:** Results of GO and KEGG enrichment analysis.

Domain	ID	Description	*P* value
BP	GO: 0031667	Response to nutrient levels	3.35*E*−19
BP	GO: 0062197	Cellular response to chemical stress	9.33*E*−19
BP	GO: 0007568	Aging	2.74*E*−18
BP	GO: 0006979	Response to oxidative stress	3.91*E*−18
BP	GO: 0033002	Muscle cell proliferation	2.50*E*−17
BP	GO: 0042493	Response to drug	1.31*E*−16
BP	GO: 0000302	Response to reactive oxygen species	4.22*E*−16
BP	GO: 0048608	Reproductive structure development	8.94*E*−16
BP	GO: 0009636	Response to toxic substance	2.80*E*−14
BP	GO: 0070482	Response to oxygen levels	5.06*E*−14
CC	GO: 0045121	Membrane raft	4.79*E*−08
CC	GO: 0098857	Membrane microdomain	4.93*E*−08
CC	GO: 0098589	Membrane region	7.07*E*−08
CC	GO: 0005635	Nuclear envelope	1.45*E*−07
CC	GO: 0031983	Vesicle lumen	5.65*E*−07
CC	GO: 0005769	Early endosome	1.79*E*−06
CC	GO: 0005667	Transcription regulator complex	3.78*E*−06
CC	GO: 0000307	Cyclin-dependent protein kinase holoenzyme complex	8.44*E*−06
CC	GO: 0090575	RNA polymerase II transcription regulator complex	8.90*E*−06
CC	GO: 0009925	Basal plasma membrane	1.68*E*−05
MF	GO: 0019902	Phosphatase binding	1.00*E*−12
MF	GO: 0019903	Protein phosphatase binding	4.10*E*−11
MF	GO: 0031625	Ubiquitin protein ligase binding	4.17*E*−07
MF	GO: 0042277	Peptide binding	5.66*E*−07
MF	GO: 0004879	Nuclear receptor activity	6.69*E*−07
MF	GO: 0098531	Ligand-activated transcription factor activity	6.69*E*−07
MF	GO: 0044389	Ubiquitin-like protein ligase binding	7.01*E*−07
MF	GO: 0140297	DNA-binding transcription factor binding	1.52*E*−06
MF	GO: 0061629	RNA polymerase II-specific DNA-binding transcription factor binding	2.11*E*−06
MF	GO: 0001223	Transcription coactivator binding	2.15*E*−06
KEGG	hsa01522	Endocrine resistance	1.79*E*−21
KEGG	hsa05219	Bladder cancer	5.14*E*−21
KEGG	hsa05215	Prostate cancer	6.44*E*−20
KEGG	hsa05212	Pancreatic cancer	8.38*E*−17
KEGG	hsa05417	Lipid and atherosclerosis	1.90*E*−16
KEGG	hsa04933	AGE-RAGE signaling pathway in diabetic complications	4.77*E*−15
KEGG	hsa05163	Human cytomegalovirus infection	8.07*E*−15
KEGG	hsa04066	HIF-1 signaling pathway	4.28*E*−13
KEGG	hsa05207	Chemical carcinogenesis—receptor activation	8.16*E*−13
KEGG	hsa04151	PI3K-Akt signaling pathway	1.78*E*−12

**Table 2:** Results of molecular docking.

Query	Core genes	PDB ID	Ingredients	LibDock score
1	PPARG	1k74 [26]	Nobiletin	84.918
2	MMP9	1gkc [27]	Nobiletin	113.515
3	JUN	1a02 [28]	Nobiletin	81.876
4	TP53	6wqx [29]	Nobiletin	80.841
5	PTGS2	5f19 [30]	Didymin	171.327
6	EGFR	1ivo [31]	Luteolin	88.705
7	MAPK3	2zoq [32]	Naringenin	82.912
8	CASP3	1cp3 [33]	Naringenin	90.267
9	AKT1	3mv5 [34]	Luteolin	102.336
10	VEGFA	1tzh [35]	Luteolin	101.616

## Data Availability

All data obtained or analyzed during this work are included within the article.

## Abbreviations

ZZP: Zhizhu pill
GERD: Gastroesophageal reflux disease
PPI: Proton pump inhibitors
CHM: Chinese herbal medicine
TCMSP: Traditional Chinese Medicine Systems Pharmacology
ROS: Reactive oxygen species.

## Supplementary Materials

Supplementary Materials Supplement A: details of the 21 active compounds and 117 targets for ZZP. Supplement B: 1476 target genes associated with GERD.
